# A mixed-methods study on end-user perceptions of transitioning to reusable surgical gowns

**DOI:** 10.1016/j.sopen.2022.10.003

**Published:** 2022-11-08

**Authors:** Ava Yap, Kaiyi Wang, Evan Chen, Caroline Melhado, Tessnim Ahmad, Patricia O'Sullivan, Seema Gandhi

**Affiliations:** a University of California San Francisco, Department of Surgery, USA; b University of California San Francisco, Office of Sustainability, USA; c University of California San Francisco, Department of Ophthalmology, USA; d University of California San Francisco, Department of Anesthesiology, USA

**Keywords:** Environmental impact, Reusable textiles, Reusable gown, Quality improvement, End user perspective, Waste reduction

## Abstract

**Background:**

Perioperative services contribute up to 70% of the US hospitals' solid waste generation. While surgical textiles are more environmentally friendly than their disposable counterparts, many US institutions have converted to disposable surgical wear in the last few decades. End-users' perception surrounding reusable textiles is currently unknown.

**Methods:**

Perioperative staff at the University of California San Francisco (UCSF) were surveyed to assess perceptions of reusable surgical gowns to guide potential implementation. The instrument included eight close-ended questions drawn from prior studies and a free-response section. The survey was piloted before dissemination. Descriptive statistics and qualitative inductive theme analysis were applied.

**Results:**

205 participants or 19.8% of the workforce responded. 77.6% perceived reusable surgical gowns as better for the environment, while 34.1% were unsure or believed that switching to reusable surgical gowns would increase surgical site infections. If given an option, 39.8% preferred reusable gowns, 30.7% preferred disposable gowns, and 25.4% had no preference. Qualitatively, four themes were identified concerning reusable gowns' 1) functionality and safety, 2) user comfort, 3) environmental concern, and 4) cost, which hindered end-user buy-in. Laundering water utilization in a drought-prone area was of particular concern.

**Conclusions:**

While most perioperative staff in a US tertiary hospital believed reusable surgical gowns were environmentally friendly, ambivalence towards transitioning to reusable gowns stemmed from uncertainty in reusable textiles' environmental benefits, safety profile, and cost savings. These perceptions may prevent successful implementation of reusable surgical gowns and suggest a need for staff education and context-specific environmental impact analyses.

**Key message:**

End-user perceptions on transitioning to reusable surgical gowns are mixed and revolve around uncertainty in their environmental benefits, cost, and functionality, which may hinder their successful implementation.

## Highlights

• Transitioning from disposable to reusable surgical gowns reduces medical waste.
• Perioperative staff perceptions are ambivalent towards reusable surgical gowns.
• Uncertainty in reusable gowns' environmental impact and effectiveness hinders buy-in.
• Closing end-users' knowledge gaps is imperative before implementing reusable gowns.

## Introduction

Despite the looming global challenge of climate change and pollution, the United States (US) healthcare system remains extremely wasteful and costly in its resource utilization [1]. With an annual healthcare expenditure reaching $3.8 trillion in 2019, the US healthcare sector ranks as the 5th largest economy in the world and is 7th in greenhouse gas emissions [2]. The perioperative service is particularly resource and energy-intensive, contributing up to 70% of solid waste generated by the US hospital system, or up to 2.8 billion pounds of waste annually [3]. Operations typically produce approximately 50 pounds of waste per case, while orthopedic and cardiac operations can produce up to 200 pounds per case [4]. Waste production in US hospitals has increased by 15% annually since 1992 largely due to increased use of disposable equipment [5].

Disposable textiles, including surgical gowns, towels and drapes, account for a significant portion of operating room waste. For more than a decade, studies have indicated that reusable textiles are an environmentally friendly alternative to their disposable counterparts. In a systematic review of five life cycle analyses (LCAs), reusable surgical gowns consistently required less energy (e.g. 4–15 megajoule [MJ] per reusable vs. 16–35 MJ per disposable), less water (e.g. 2.9 gallons per reusable vs. 3.7 gallons per disposable), and produced a carbon footprint that was 2–3 times smaller than disposable surgical gowns [6]. In this review, cost studies comparing reusable and disposable textiles demonstrated mixed results. More recently, another LCA demonstrated that reusable surgical gowns reduced natural resource energy consumption by 64%, greenhouse gas emissions by 66%, blue water conservation by 83% and solid waste generation by 84% [7]. Furthermore, reusable gowns have been shown to reduce costs of waste disposal [4]. In their 2019 guidelines for perioperative practices, the Association of Perioperative Registered Nurses (AORN) declared reusable equipment and supplies as the preferred choice to reduce waste [8].

Nevertheless, many US healthcare institutions and networks remain heavily or exclusively reliant on disposable surgical textiles, and 80% of the healthcare textile market consists of disposable materials [9]. Given the growing evidence in support of reusable textiles, guidelines have been issued outlining implementation of reusable gowns, with an emphasis on understanding the operating room (OR) culture to facilitate change [10]. However, no previous study has assessed attitudes towards reusable surgical textiles in the context of high disposable textile utilization. The purpose of this study was to determine the perceptions of the perioperative staff – including attending surgeons, trainee surgeons, and OR staff – on the adoption of reusable surgical gowns.

## Methods

### Overall design

This was a cross-sectional study utilizing a survey comprising of close-ended questions and a free-response section, designed to obtain perspectives from perioperative staff regarding reusable surgical gowns during the month of September 2021, following the Strengthening the Report of Observational Studies in Epidemiology (STROBE) guidelines (see checklist in Appendix A). The study was reviewed and approved as exempt by the UCSF Institutional Review Board.

### Study setting and participant recruitment

UCSF is a large tertiary medical and surgical healthcare system and surgical hub that exclusively uses disposable textiles in the operating rooms, including gowns, drapes, towels, and back table and mayo stands covers. An internal audit found that textiles comprise 40% of landfill waste from surgical cases. Perioperative staff members at UCSF campuses received an institutional email to voluntarily participate in the survey. Relevant staff positions included attending surgeons and anesthesiologists, surgical and anesthesia post-graduate trainees (residents and fellows), and operating room (OR) staff (including scrub technologists and circulating nurses). Anesthesiologists were eligible participants because they were also important end-users of the reusable gowns, donning them during perioperative sterile procedures such as central line placements, which are especially common given the center's large number of transplants and other high-risk cases.

### Survey instrument

Questions were informed by previous research in reusable perioperative equipment consisting of qualitative and performance studies [[11], [12], [13]]. Based on literature review, close-ended questions revolved around participants' perceptions on 1) environmental implications, 2) sterility, 3) comfort, and 4) cost savings. To measure participant experience in their job, we categorized their career duration based on average length of an academic faculty position from assistant to full professorship, given that this study was conducted in an academic setting [25,26]. Additionally, a free response section allowed for inclusion of additional comments. The questionnaire was piloted on a small group (n = 10) of surgical and anesthesia trainees who provided feedback to ensure clarity, internal validity, and ease of use. Participants received a link to an anonymous online survey using the Qualtrics platform (Qualtrics, Provo, UT). The survey remained open during the month of September 2021. During this period, a second reminder email the same link was distributed to solicit further responses.

### Qualitative analysis

Four study researchers (3 surgical trainees, 1 sustainability analyst) qualitatively analyzed, via inductive thematic analysis, all free-text responses to the survey. Each member developed preliminary descriptive codes that reflected perceptions on surgical textiles through an immersion and crystallization process. The researchers then met to refine the codebook into a relevant list of codes including concerns of effectiveness, sterilizability, infection concern, comfort, quality maintenance, ambiguity in environmental impact of reusable products, perception of reusable and/or disposable gowns having an adverse effect on the environment, disposable and/or reusable gown costs, and prior experience using reusables. The codebook is showcased in Appendix Table 1. Coding discrepancies were resolved via consensus and the round of final analysis consolidated the codes into four overarching themes. Exemplary quotes (with RXX indicating the specific respondent) were selected to represent each theme.

### Statistical analysis

Close-ended, multiple choice survey questions were analyzed using chi- squared or Fisher exact tests when categories frequencies are smaller than 20 respondents when stratified by role (i.e., attending, trainee, OR staff) and years of practice. Missing values were excluded for analysis. P-values less than 0.05 were considered statistically significant, though trends towards significance are also mentioned as the small number in some subgroups in analysis confers a higher possibility for a type II error. The analyses were performed using R software, version 4.1.1 “Kick Things” (The R Foundation for Statistical Computing, Vienna, Austria).

## Results

### Quantitative results

Two hundred and five perioperative staff members or 19.8% of the workforce responded to the survey. Among the respondents, 47.3% (n = 97) were surgical or anesthesia attendings, 36.1% (n = 74) were surgical or anesthesia trainees, and 16.6% (n = 34) were OR staff, including OR nurses and scrub technologists, 47.3% (n = 97) of the respondents were male and a plurality were between the ages 31–40 years old (34.6%, n = 71). 35.1% (n = 72) of the respondents were anesthesia attendings or trainees. 38.5% (n = 79) have held their current job for less than 5 years. Within the surgeon respondents, general surgery (16.1%, n = 33) and otolaryngology (9.3%, n = 19) were most represented. Overall, 76.6% (n = 157) of respondents perceived reusable surgical gowns as more environmentally friendly, and 53.2%, (n = 109) perceived single-use gowns as more costly. Table 1 showcases the breakdown of responses on the questions regarding reusable surgical gown beliefs. Among all the respondents, 50 (24.4%) provided comments for the qualitative analysis.

**Table 1:** Participant characteristics and responses for the close-ended questions regarding beliefs and preferences surrounding reusable surgical gowns.

Overall (N = 205)	Frequency	Percentage
Position		
Attending	97	(47.3%)
OR staff	34	(16.6%)
Trainee	74	(36.1%)
Surgery or anesthesia staff		
Surgery	99	(48.3%)
Anesthesia	72	(35.1%)
Sex		
Female	100	(48.8%)
Male	97	(47.3%)
Prefer not to say	8	(3.9%)
<5	79	(38.5%)
5–10	55	(26.8%)
11–20	38	(18.5%)
>20	32	(15.6%)
No response		
Opinion on which product is more environmentally friendly		
Disposable surgical gowns	10	(4.9%)
Neither	34	(16.6%)
Reusable surgical gowns	157	(76.6%)
No response		
Opinion on which product is more costly		
Disposable surgical gowns	109	(53.2%)
Neither	30 (14.6%)	
Reusable surgical gowns	60 (29.3%)	
Gown preference if given both options		
Disposable surgical gowns	62 (30.2%)	
I do not have a preference	52 (25.4%)	
Reusable surgical gowns	89 (43.4%)	
No response	2 (1.0%)	
Belief that reusable gowns increase surgical site infections		
No	132 (64.4%)	
Unsure	53 (25.9%)	
Yes	18 (8.8%)	
Missing	2 (1.0%)	

60.5% (n = 124) of the respondents believed that operating rooms should provide both reusable and single-use surgical gowns options. If both options of single-use and reusable surgical gowns were comparable and available, there was a significant difference between attendings, staff and trainees with more attendings and trainees preferring the reusable gown option (p = 0.002, Table 2). Compared to trainees and attendings, OR staff were more likely to think that the transition to reusable gowns could lead to higher SSI rates, though this finding trended towards but did not reach statistical significance (p = 0.058). When stratified by years of practice, respondents who were in practice over 20 years were more frequently believed that there is no link between the use of reusable surgical gowns and increase in SSI, and thus prefer the reusable gowns over the single-use gowns option, though statistical significance was not reached (Table 3). There was also no difference among surgeons and anesthesia respondents in terms of which gowns they prefer (p = 0.326) (Table 4).

**Table 2:** Attendings, trainee and OR staff perceptions on transitioning to reusable surgical gowns.

	Surgery or anesthesia attending	OR staff	Surgery or anesthesia trainee	p-Value
*Would transition to reusable gowns lead to increase in SSI?*				
No	71 (74.0%)	17 (50.0%)	44 (60.3%)	0.058
Unsure	19 (19.8%)	11 (32.4%)	23 (31.5%)	
Yes	6 (6.6%)	6 (17.6%)	6 (8.2%)	

*Which gown would you prefer to use, if available?*				
Single-use surgical gowns	25 (26.0%)	20 (58.8%)	17 (23.3%)	0.002
No preference	24 (25.0%)	4 (11.8%)	24 (32.9%)	
Reusable surgical gowns	47 (49.0%)	10 (29.4%)	32 (43.8%)

**Table 3:** Staff perceptions on reusable gowns stratified by years in practice.

	In practice ≤ 20	In practice > 20	p-Value
*Would transition to reusable gowns lead to increase in SSI?*			
No	109 (62.6%)	24 (77.4%)	0.306
Unsure	48 (27.6%)	6 (19.4%)	
Yes	17 (9.9%)	1 (3.1%)	

*Which gowns do you prefer?*			
Disposable surgical gowns	55 (32.0%)	8 (25.0%)	0.460
I do not have a preference	45 (26.2%)	6 (18.8%)	
Reusable surgical gowns	72 (41.9%)	18 (56.3%)

**Table 4:** Preference of reusable or disposable gowns between anesthesia and non-anesthesia respondents.

	Anesthesia (N = 72)	Non-anesthesia (N = 133)	p-Value
*Which gowns do you preferred*			
Disposable surgical gowns	45 (34.1%)	17 (23.9%)	0.326
I do not have a preference	32 (24.2%)	20 (28.2%)	
Reusable surgical gowns	55 (41.7%)	34 (47.9%)	

### Qualitative results

#### Overview

For the qualitative analysis of the survey, twelve codes were identified in participant responses, which were further consolidated into four overarching themes on staff attitudes towards the transition to reusable surgical gowns. These responses centered around reservations participants had that may preclude them from adopting reusable gowns. Some also welcomed the initiative, lauding the reusable gown as an environmentally conscious alternative to the current disposable option. Main issues with reusable gown implementation that arose in the comments revolved around 1) concerns of adequate functionality and safety profile, 2) user comfort, 3) uncertainty and interest in minimizing product environmental impact, balanced with 4) cost concerns (Table 5). A consensus to select the gown that was more environmentally friendly and less costly permeated across all staff positions and age groups, but equipoise lingered on whether the disposable or reusable gown met these criteria. Participants brought up previous anecdotal experiences with reusable gowns to substantiate their opinions. Given the uncertainty, there was a call for more investigation in the field. Furthermore, many proposed keeping both gown options available for staff to select based on their preferences.

**Table 5:** Theme descriptions of end-user perceptions on reusable gowns, with exemplary supporting quotes.

Theme	Description	Example quotes
Functionality and safety	Efficacy in serving the gown's purpose as a sterile barrier. Considerations include infection control, safety profile, impermeability, quality maintenance	“I feel that reusable gowns really involve a layer of trust that the gowns truly are disinfected and that dirty gowns aren't accidentally incorporated into the pile of clean gowns. Having used reusable gowns in the ICU and floor I know that it is not always clear if a gown is clean. Disposable gowns must be physically opened and are sterile at the point of opening. In the operating room, we take sterility extremely seriously and I would personally not use a reusable gown in the OR because I can't truly be certain it is sterile to the same degree a disposable gown is.” (R38) “I believe there will have to be a cultural change and evidence-based data to convince staff members to use reusable gowns during surgery - since sometimes the scrubs we are provided come back with stains etc.” (R47)
User comfort	End-user concerns about wearability and breathability, especially over a longer duration	“Some reusable gowns have ventilation issues and can be incredibly stifling. This is one area where staff buy-in will be incredibly difficult.” (R12) “Some people prefer the paper gowns as they are lighter and cooler, and it is a nice option if you are wearing lead, pregnant, or otherwise in a room requiring high heat or layers.” (R48)
Environmental concern	Ambiguity around environmental benefits and consequences of both reusable and disposable gowns, with special attention to water usage in a drought-prone state	“I have no idea which is more environmentally friendly... To me, this is a data free zone though my guess is that there is data and I just don't know what it is (!) happy to do whatever is safe but also makes sense for our world.” (R24) “I would use whatever is assessed to be more environmentally friendly… If people have a misconception just educate. (R13)” “Many surgeons scrub in and out 5–10 times a case. This creates a huge amount of waste” (R11). “Are reusable surgical gowns really that more environmentally friendly? Water to wash them? Detergent? Aren't we in a drought?” (R36)
Cost	Concerns about the comparative costliness between the utilization and processing of reusable and disposable gowns.	“I know that the costs of laundering can be very high. If thinking about the environment and not cost, reusable makes sense. If considering cost, I don't know which is less expensive.” (R50) “Over the years (…) cost is usually the driving factor. To hire an outside company to clean/sterilize gowns is far more costly than the disposable gowns.” (R07)

#### Functionality and safety

Concerns about the integrity of the reusable gowns after multiple uses left some respondents unsure about its sterility and safety profile, particularly for high-stakes surgical cases, such as trauma laparotomies with large volume blood loss, or neurosurgical and orthopedic procedures involving implants. These cases are thought to generally require reinforced or impervious “level 4” gowns, as graded by the Association of the Advancement of Medical Instrumentation (AAMI). Some thought that reusable gowns should be reserved for lower risk surgeries or for personnel that were not “quite as active in the field” (R34), while disposable gowns were perceived as superior in sterility due to single utilization. In terms of quality maintenance, Durability of flimsier disposable gowns was questioned, though potential for reusable gown tears after repeated uses were also a concern. Some were critical of disposable gowns' susceptibility to tears and lauded reusable gowns as being more “impermeable” (R30). Poor experiences from other (albeit non-sterile) reusable medical wear drove the apprehension and mistrust towards reusable surgical gowns.

#### User comfort

Some respondents raised concerns about the comfort and wearability of the reusable gowns. While respondents stated that gowns, either disposable or reusable, can be uncomfortable due to overheating and sizing, most stated that they would prefer the more comfortable option. Opinions again seemed to be based on prior experiences. Qualities that the end-users valued included breathability, weightlessness, and cooling capacity.

#### Environmental concern

While most respondents preferred more environmentally friendly products, they were unsure whether the reusable or disposable gowns were better for the environment. Some respondents suggested they would make the switch if reusables were indeed more environmentally friendly but conceded that they lacked the knowledge to commit to the transition. One individual suggested further research and re-education of the peri-operative community is needed. Others had strong opinions about the wasteful nature of disposable gowns, especially revolving around surgeon practices during long cases with multiple episodes of donning and doffing gowns. Meanwhile, respondents were also dubious about reusable gowns' environmental impact from laundering and re-sterilization with its accompanying water, detergent, and electricity usage. The concern about water utilization was especially salient given the perpetual drought conditions in California.

#### Cost

Related to resource utilization, costs of gown usage were also an important consideration for many respondents. Participants did not know the balance between costs incurred from disposable and reusable gowns, which left many unsure on a preferred gown. Several respondents were concerned about the possibility of increased cost associated with laundry and sterilization for reusable gowns, positing that these may be higher than the manufacturing costs of disposable gowns. Notably, no responses mentioned cost concerns regarding the disposable gowns.

## Discussion

The current US healthcare system relies heavily on disposable supplies, and is the second largest contributor of waste in the United States after the food industry [4]. While growing evidence has demonstrated that reusable textiles have significantly reduced carbon footprint, energy consumption, emissions, water use and waste generation compared to single-use options in multiple settings [6,11,[14], [15], [16], [17]], the majority of the healthcare market continues to utilize disposable textiles. Gown end-users' perceptions and beliefs about reusable options have not yet been investigated. In this study, perioperative staff in a Californian tertiary hospital system that solely utilizes disposable surgical textiles were surveyed about potentially transitioning to reusable surgical gowns. Consistent themes across responses included competing concerns and uncertainties about safety, comfort, environmental impact, and cost. Respondent comments suggested a dearth of knowledge in these domains which precluded informed comparison between reusable and disposable gowns and comfortable adoption of the latter. Overall, our findings identified potential barriers to reusable surgical gown adoption among perioperative staff, and the need for further probing studies on how best to address these uncertainties prior to reusable gown implementation.

Nearly a quarter of the perioperative staff who responded were hesitant to adopt reusable gowns, citing belief of steep laundering costs and questionable environmental impact. In the context of previously discussed research, these responses were incongruent with the overall favorable environmental profile of reusable gowns. Additionally, some survey respondents expressed concern that institutional and geographic-specific factors, such as supply chains, climate, and drought conditions, may exacerbate the environmental impact and cost of reusable options. Therefore, accurate appraisal of reusable versus disposable gowns' environmental impact would need to bolster pre-existing LCA studies with an institution specific LCA that accounts for the site's unique characteristics.

Many respondents expressed concern that sustainability driven goals would compromise functionality, though this is not supported in prior studies that demonstrate noninferiority of reusable gowns in metrics such as tear strength, sterility, and water resistance [18,19]. Current reusable gowns on the market are also approved by the Food and Drug Administration (FDA) guidelines and meet AAMI barrier standards [20], while industrial laundering compliant with Center of Disease Control (CDC) standards should not compromise reusable gowns' barrier protection performance [19]. Reusable gowns have also not been associated with higher rates of surgical site infection [13,21]. Therefore, providing detailed information through staff education about reusable's gowns prevailing functionality over repeated laundering cycles could be effective in addressing healthcare workers concerns about these factors.

Concerns about the comfort and breathability of reusable gowns for end-users have stymied prior implementation in some older studies have using reusable gown options which were significantly less air permeable [19]. Nevertheless, a study of reusable gown adoption at the University of California Los Angeles found that initial complaints of discomfort diminished as staff became accustomed to them [22]. At another institution, staff adherence in isolation gown wear did not differ in reusable or disposable gown material when accounting for fit or comfort issues [23]. Another study involving surgeons and surgical technicians rated reusable gowns as more comfortable than disposable ones [4]. These results indicate that initial concerns about discomfort may not be a prohibitive barrier to utilization, and exposure to gown options prior to implementation may help with acceptance.

Disposable wear is inherently unsustainable, as exemplified by the height of the COVID-19 pandemic when severe shortages in personal protective equipment (PPE) rapidly manifested and accelerated waste accumulation [18]. In an effort to ameliorate PPE shortages during the pandemic, our institution increased the use of reusable isolation gowns as recommended by the CDC [24]. Unfortunately, prior negative experiences with these non-surgical isolation gowns spurred poor perceptions of reusable gowns in general. Therefore, it is important during reusable equipment initiatives to distinguish quality of surgical wear from that of other hospital garments to avoid transference. Implementation of reusable medical equipment can be optimized by clarifying the specific purpose of each item as well as its differences to prior, similar equipment.

Operating room staff such as circulating nurses and scrub technologists were noted to be more hesitant to embrace reusable surgical gowns when compared to attendings and trainees. We posit that the transition to reusable products could add or disrupt their well-established workflow. Furthermore, OR staff are often responsible for the sterility of equipment and surgical textiles, including preparation of the surgical back-table. Lack of knowledge and trust of gown sterility may exacerbate utilization reluctance. Engagement with perioperative staff and obtaining their input on gown implementation is imperative to ensure buy-in and successful uptake of reusable surgical gowns. Furthermore, special attention must be paid to engage scrub technicians and circulating nurses in the endeavor, as they are key stakeholders in the gown utilization workflow but have more ambivalence towards reusable gowns' effectiveness and environmental benefits.

This paper has several limitations. First, our survey responses are derived from the single medical system of UCSF in the city of San Francisco, which may not be widely generalizable to other healthcare settings. This may be especially pertinent given the study's setting within a drought-prone state. Nevertheless, our findings are reflective that context-specific environmental concerns should be accounted for when and where one is considering implementing reusable surgical gowns. Secondly, responses may be subject to recall bias as a self-reported questionnaire without proctorship, though this hood of anonymity might also allow for more transparent commentary. Thirdly, generalizability may also be limited by low response rate. Nevertheless, the proportionality across respondent position (i.e. surgical, anesthesia and perioperative staff) and facilities reflects the distribution is likely representative of the broader target population. Relatedly, respondents may have been more likely to express strong preferences about reusable surgical gowns compared to nonrespondents as is common in volunteer-based surveying techniques.

## Conclusion

Sustainability and supply chain resilience have become increasingly important topics for the healthcare industry. Single-use surgical products comprise a substantial proportion of opportunities for carbon emissions, waste generation, and resource utilization. Considerable evidence has already demonstrated that reusable surgical gowns have a lesser environmental impact with comparable or superior functionality. In this study, most respondents expressed interest in reusable surgical products. However, nearly a quarter were hesitant about adoption and expressed concern that reusables may not yield lower environmental impact. Understandably, awareness of these studies has not yet permeated among all healthcare workers and the adoption of reusable products from the standpoint of perioperative staff may be limited by an existing knowledge gap. Our study demonstrates the importance of education and workflow optimization in implementation efforts along with a potential need for institution-specific life-cycle analysis to affirm the lesser environmental impacts from the reusable surgical gowns.

## Funding sources

This was an unfunded study and was conducted with institutional resources which are freely available to all staff members. This research did not receive any specific grant from funding agencies in the public, commercial, or not-for-profit sectors.

## Ethics approval

This study protocol was reviewed by the University of California San Francisco Institutional Review Board and was deemed to be exempt from requiring approval.

## CRediT authorship contribution statement

AY, KY, and SG formulated the conception of the study. AY and KY performed the data curation and acquisition. AY, KW, EC, CM, TA, and PS contributed to the study design and analysis. PS provided expert guidance and methodological validation. All authors participated in the drafting, editing, and critical review of the manuscript.

## Conflict of interest

The authors do not have any relevant conflicts of interest to disclose. The authors report no proprietary or commercial interest in any product mentioned or concept discussed in this article.

## Figures and Tables

**Table 1:** Codes used for the qualitative analysis portion of the study.

Number	Codes	Description
1	Concern of effectiveness	Concerns of efficacy on reusable equipment fulfilling their purpose and functionality to be non-inferior to disposable counterparts or vice-versa concern for effectiveness of reusable vs disposable were coded separately.
2	Sterilizability	Concerns that the reusable material will need to be adequately cleaned to be safe for subsequent use
3	Infection concern	Concern about surgical site infections related to reusable equipment
4	Comfort	Concern about comfort, temperature control, wearability of the alternative reusable gowns (vs. standard disposable)
5	Quality maintenance	Concern for storage, supply and maintenance.
6	Ambiguity in environmental impact of reusable products	Demonstration of lack of knowledge or import in equipment's environmental impact
7	Perception of reusable gowns having adverse effect on the environment	Concern for reusable resources having a large carbon footprint, waste creation and water utilization
8	Perception of disposable gowns having adverse effect on the environment	Concern for disposable resources having a large carbon footprint, waste creation and water utilization
9	Disposable gown costs	Concern for cost of production and disposal
10	Reusable gown costs	Concern for cost of production, maintenance and laundering
11	Prior experience using reusable gowns	Demonstration of opinion from prior facility or work
12	Both should be offered	Concern that specific cases necessitate reusable vs disposable

**Table 2:** STROBE Statement—checklist of items that should be included in observational studies.

	Item #	Recommendation	Included
Title and abstract	1	(a) Indicate the study's design with a commonly used term in the title or the abstract	✔
		(b) Provide in the abstract an informative and balanced summary of what was done and what was found	✔
Introduction			
Background/rationale	2	Explain the scientific background and rationale for the investigation being reported	✔
Objectives	3	State specific objectives, including any prespecified hypotheses	✔
Methods			
Study design	4	Present key elements of study design early in the paper	✔
Setting	5	Describe the setting, locations, and relevant dates, including periods of recruitment, exposure, follow-up, and data collection	✔
Participants	6	(a) *Cohort study*—Give the eligibility criteria, and the sources and methods of selection of participants. Describe methods of follow-up *Case–control study*—Give the eligibility criteria, and the sources and methods of case ascertainment and control selection. Give the rationale for the choice of cases and controls *Cross-sectional study*—Give the eligibility criteria, and the sources and methods of selection of participants	✔
		(b) *Cohort study*—For matched studies, give matching criteria and number of exposed and unexposed *Case–control study*—For matched studies, give matching criteria and the number of controls per case	N/A
Variables	7	Clearly define all outcomes, exposures, predictors, potential confounders, and effect modifiers. Give diagnostic criteria, if applicable	✔
Data sources/measurement	8	For each variable of interest, give sources of data and details of methods of assessment (measurement). Describe comparability of assessment methods if there is more than one group	✔
Bias	9	Describe any efforts to address potential sources of bias	✔
Study size	10	Explain how the study size was arrived at	✔
Quantitative variables	11	Explain how quantitative variables were handled in the analyses. If applicable, describe which groupings were chosen and why	✔
Statistical methods	12	(a) Describe all statistical methods, including those used to control for confounding	✔
		(b) Describe any methods used to examine subgroups and interactions	✔
		(c) Explain how missing data were addressed	✔
		(d) *Cohort study*—If applicable, explain how loss to follow-up was addressed *Case–control study*—If applicable, explain how matching of cases and controls was addressed *Cross-sectional study*—If applicable, describe analytical methods taking account of sampling strategy	N/A
		(e) Describe any sensitivity analyses	N/A
Results			
Participants	13*	(a) Report numbers of individuals at each stage of study—e.g. numbers potentially eligible, examined for eligibility, confirmed eligible, included in the study, completing follow-up, and analyzed	✔
		(b) Give reasons for non-participation at each stage	N/A
		(c) Consider use of a flow diagram	N/A
Descriptive data	14*	(a) Give characteristics of study participants (e.g. demographic, clinical, social) and information on exposures and potential confounders	✔
		(b) Indicate number of participants with missing data for each variable of interest	✔
		(c) *Cohort study*—Summarise follow-up time (e.g., average and total amount)	N/A
Outcome data	15*	*Cohort study*—Report numbers of outcome events or summary measures over time	N/A
		*Case–control study*—Report numbers in each exposure category, or summary measures of exposure	N/A
		*Cross-sectional study*—Report numbers of outcome events or summary measures	✔
Main results	16	(a) Give unadjusted estimates and, if applicable, confounder-adjusted estimates and their precision (e.g., 95% confidence interval). Make clear which confounders were adjusted for and why they were included	N/A
		(b) Report category boundaries when continuous variables were categorized	✔
		(c) If relevant, consider translating estimates of relative risk into absolute risk for a meaningful time period	N/A
Other analyses	17	Report other analyses done—e.g. analyses of subgroups and interactions, and sensitivity analyses	✔
Discussion			
Key results	18	Summarise key results with reference to study objectives	✔
Limitations	19	Discuss limitations of the study, taking into account sources of potential bias or imprecision. Discuss both direction and magnitude of any potential bias	✔
Interpretation	20	Give a cautious overall interpretation of results considering objectives, limitations, multiplicity of analyses, results from similar studies, and other relevant evidence	✔
Generalisability	21	Discuss the generalisability (external validity) of the study results	✔
Other information			
Funding	22	Give the source of funding and the role of the funders for the present study and, if applicable, for the original study on which the present article is based	✔

Note: An Explanation and Elaboration article discusses each checklist item and gives methodological background and published examples of transparent reporting. The STROBE checklist is best used in conjunction with this article (freely available on the Web sites of PLoS Medicine at http://www.plosmedicine.org/, Annals of Internal Medicine at http://www.annals.org/, and Epidemiology at http://www.epidem.com/). Information on the STROBE Initiative is available at www.strobe-statement.org.
